# Use of Sodium Butyrate as an Alternative to Dietary Fiber: Effects on the Embryonic Development and Anti-Oxidative Capacity of Rats

**DOI:** 10.1371/journal.pone.0097838

**Published:** 2014-05-22

**Authors:** Yan Lin, Zheng-feng Fang, Lian-qiang Che, Sheng-yu Xu, De Wu, Cai-mei Wu, Xiu-qun Wu

**Affiliations:** Key Laboratory for Animal Disease Resistance Nutrition of the Ministry of Education, and Animal Nutrition Institute of Sichuan Agricultural University, Ya'an, Sichuan, P R China; Massachusetts General Hospital, United States of America

## Abstract

In this study, we evaluated the effect of replacing dietary fiber with sodium butyrate on reproductive performance and antioxidant defense in a high fat diet during pregnancy by using a rat model. Eighty virgin female Sprague Dawley rats were fed one of four diets—(1) control diet (C group), (2) high fat + high fiber diet (HF group), (3) high-fat +5% sodium butyrate diet (SB group), and (4) HF diet + α-cyano-4-hydroxy cinnamic acid (CHC group)—intraperitoneally on days 8, 10, 12, 14, and 16 of gestation. SB and dietary fiber had similar effects on improving fetal number and reducing the abortion rate; however, the anti-oxidant capacity of maternal serum, placenta, and fetus was superior in the HF group than in the SB group. In comparison, CHC injection decreased reproductive performance and antioxidant defense. Both dietary fiber (DF) and SB supplementation had a major but different effect on the expression of anti-oxidant related genes and nutrient transporters genes. In summary, our data indicate that SB and DF showed similar effect on reproductive performance, but SB cannot completely replace the DF towards with respect to redox regulation in high-fat diet; and SB might influence offspring metabolism and health differently to DF.

## Introduction

Inadequate fiber intake by humans is widespread, especially in developed countries [1]. Addition of dietary fiber to a high-fat, Western-style diet has beneficial effects on both maternal and fetal health [2]. Experimental investigations indicate that supplementing high-fat or low-fat diets with fiber might promote embryo survival and fetal development [3], [4] and reduce the oxidative stress of the organism [5], thus improving offspring health [6]. However, the mechanisms that cause maternal fiber intake to have a positive effect on the development and health of offspring have yet to be elucidated.

However, a large number of short chain fatty acids (SCFAs), including acetic, propionic, and butyric acid, are formed during bacterial fermentation in the gut after fiber intake. The colonic absorption of SCFAs might provide energy sources for colonocytes, liver and peripheral tissues [7]. The relative contribution of SCFAs to metabolic requirements differs across various mammals, being 10% in humans, 30–40% in typical hindgut fermenters, and 80% in forestomach fermenters [8]. In mammals, acetic, propionic, and butyric acid are the main components of SCFAs, with an energy supplement ranking of butyrate > propionate > acetate and butyric acid having the strongest nutritional effects [9], [10]. SCFAs that escape from colon cells enter the portal vein of the liver and can be converted into acetyl coenzyme A in the liver [11], which is then used in many biochemical reactions. This information indicates that SCFAs might contribute to the physiological effects of fibers in the body, especially butyrate. Previous studies have shown that butyrate improves the development of cloned porcine embryos in vitro [12], provides beneficial effects on the in vitro developmental competence of porcine somatic cell nuclear transfer [13], and activates genes involved in early development [14]. In vivo data indicate that orally administered sodium butyrate (SB) promotes the growth performance of weaning pigs [15], improves the intestinal health of humans [16], and delays the onset of diabetes in rats [17]. SB supplementation has a similar effect to wheat bran and guar gum on antioxidant property in diabetic rats [18]. Other studies have shown that diets supplemented with single SCFA or SCFA mixture may have a similar role to sugar-beet fiber in lowering the plasma cholesterol of rats [19] and alleviating rat colitis [20]. Therefore, it is plausible to hypothesize that the administration of butyrate might have the same effects as dietary fiber on maternal and fetal health. However, relatively few studies have reported the effect of maternal SB intake on embryo development, anti-oxidant capacity, and the gene expression of offspring.

Recent studies have demonstrated that the monocarboxylate transporter (MCT) is involved in the absorption of SCFAs across the apical membrane in both humans [21] and rats [22]. Fourteen MCT isoforms have been identified to date in mammals. MCT1 and MCT2 have very similar substrate specificities, but they differ with respect to affinity. MCT4, on the other hand, seems to be adapted for highly glycolytic cells, it is involved in the removal of lactic acid produced from glycolysis [23]. The expressions of MCT1, MCT4 and MCT9 mRNA increased significantly in mouse placenta from gestational day 11.5 and decreased at the end of gestation [24]. A significant increase in the expression levels of MCTs was observed in the rat adrenal gland after pectin supplementation [25], while butyrate and lactate increased the expression and activity of MCT1 in vitro [26], [27]. Thus, MCTs might be involved in the regulation of dietary fiber during reproduction. However, information remains limited about the effect of fiber intake on the expression of MCTs in the placenta or whether the inhibition of MCT expression alters the action induced by dietary fiber. Therefore, in this study, we aimed to determine whether supplementation with SB during pregnancy has a similar effect to fiber in improving fetal development and ameliorating maternal and placenta oxidative stress induced by a high-fat diet. In addition, we investigated how MCT expression during embryo development and reproduction is inhibited by injecting the MCT inhibitor a-cyano-4-hydroxycinnamic acid.

## Materials and Methods

### Animals and Diets

Eighty virgin female Sprague Dawley rats were offered a standard laboratory chow diet (20% protein) and water ad libitum until pregnancy was confirmed through the observation of vaginal plugs. Pregnant rats were allocated into 4 groups: (1) control diet (C), (2) high-fat + high-fiber diet (HF), (3) high-fat and sodium butyrate diet (SB, 5%, Purity ≥98.5% Sigma-Aldrich, St. Louis, MO, USA), and (4) high-fat and high-fiber diet with the injection of α-cyano-4-hydroxy-cinnamic acid (CHC, Sigma C2020, Purity ≥98%). The compositions of the experimental diets are presented in Table 1. Fat was composed of soybean oil, rapeseed oil, and pig lard, with fiber being provided by oat and wheat bran at a ratio of 1∶1. Fresh diets were made up on a weekly basis, and all diets were stored at 4°C. Animals were then housed separately at a temperature of 18–26°C on a 12-h light/12-h dark cycle, and they had free access to food and water until sample collection was performed on day 19.5 of pregnancy. All experimental protocols were approved by the Animal Care and Use Committee of the Sichuan Agricultural University, China, and were in accordance with the National Research Council's Guide for the Care and Use of Laboratory Animals.

**Table 1:** Compositions of experimental diets based on the AIN-93 diet.

Ingredient (g/kg DM)	C	HF	SB	CHC
Corn starch	55.05	20.90	37.05	20.90
Wheat bran	0.00	12.50	0.00	12.50
Oat	0.00	12.50	0.00	12.50
Casein	20.00	16.15	20.00	16.15
Sucrose	10.00	10.00	10.00	10.00
Soybean oil	7.00	10.00	10.00	10.00
Lard	0.00	10.00	10.00	10.00
Vitamin mixture	1.00	1.00	1.00	1.00
Mineral mixture	3.50	3.50	3.50	3.50
L-cysteine	0.20	0.20	0.20	0.20
Cellulose	3.00	3.00	3.00	3.00
Choline bitartrate	0.25	0.25	0.25	0.25
Sodium butyrate	0.00	0.00	5.00	0.00
Total	100.00	100.00	100.00	100.00
Gross energy (KJ/g)	19.50	22.16	22.69	22.16
Protein (%)	17.00	17.00	17.00	17.00
Fat (%)	7.00	20.00	20.00	20.00
Fiber(%)	3.00	9.54	3.00	9.54

C, control group; HF, high-fat and high-fiber group; SB, high-fat and sodium butyrate group; CHC, HF diet with CHC injection group.

### Intraperitoneal CHC Injection

Embryo implantation occurs on about the 7th day of pregnancy in rats [28]. Rats in the CHC group were injected intraperitoneally with CHC on days 8, 10, 12, 14, and 16 of pregnancy, while the rats in the other groups were injected with saline on the same days, to serve as controls.

### Blood and Tissue Sample Collection

Before sample collection, the rats were anesthetized in a sealed jar containing cotton wetted with 350 µl of isoflurane. Blood was drawn from the jugular vein and centrifuged at 4000 rpm for 10 min at 4°C for serum collection. Serum was stored at −20°C until analysis. The placenta and fetus were then immediately removed, weighed, and stored at −70°C until analysis. Part of the placenta and fetal liver were used for the histological examination. Fresh cecum content was collected from each rat and immediately processed for the SCFA analysis.

### Measurements and Analytical Methods

#### Histological examination

Small 0.5×0.5-cm segments of placental and fetal liver were cut and rinsed with ice-cold phosphate buffer saline. The segments were then fixed in 10% neutral buffered formalin, routinely processed, and stained with hematoxylin and eosin for histological examination.

#### SCFA analysis

SCFA concentrations in the cecum contents were evaluated after extraction by using a previously described method [29]. Individual SCFAs were measured with a gas chromatograph (CP-3800, Varian, Lake Forest, CA, USA) equipped with a flame ionization detector and a capillary column (25 mm×0.32 mm, 0.3-µm film thickness; Varian Inc., USA). Helium was used as the carrier gas, and isobutyric acid was used as an internal standard. A standard SCFA mixture containing acetate, propionate, and butyrate was used for the calculation, and the results were expressed as mmol/g of sample.

#### Assay of malonaldehyde (MDA) and protein carbonyl concentrations

Before the assays, placenta and fetal liver were homogenized in 1 ml of 0.15 M NaCl solution and centrifuged at 3000 rpm for 10 min at 4°C. Then, the supernatant was used for the analysis. The MDA concentrations in the serum, placenta, and fetus liver were quantified using the the thiobarbituric acid method [30], which is based on the reaction of MDA with thiobarbituric acid to form a pink chromogen. Protein carbonyl levels were determined from reactions with dinitrophenylhydrazine, using a commercially available kit (Oxiselect protein carbonyl ELISA; Cell Biolabs, San Diego, CA, USA), according to the manufacturer's instructions. Absorbance was measured at 370 nm, and concentrations were calculated using a molar extinction coefficient of 22,000 M/cm at 370 nm after the subtraction of the blank absorbance. Data were normalized to protein content and expressed as nmol/mg protein.

#### Assay of anti-oxidant related enzyme activity

Glutathione peroxidase (GPx), catalase (CAT), total superoxide dismutase (T-SOD), Mn-SOD, and Cu-Zn SOD activity in maternal serum, placenta, and fetal liver were determined using a commercially available kit (Nanjing Jiancheng Bioengineering Institute, Nanjing, China), following the manufacturer's protocols. All assays were carried out in triplicate, using samples from six different rats per group. One unit of CAT activity was defined as 1 µM of H_2_O_2_ consumed per min, whereby 1 unit of GSH-Px was defined as the amount of enzyme necessary to oxidize 1 µmol/NADPH/min at pH 7.0 at 25°C and 1 unit of T-SOD activity was defined as the amount of sample protein capable of inhibiting the reduction of NBT by 50% of maximum inhibition. Mn-SOD activity was measured by the addition of 5 mmol/l NaCN to the reaction mixture, while Cu-Zn SOD was calculated by subtracting the Mn-SOD value from T-SOD. Enzyme-specific activities were expressed as units/mg of protein. Total protein concentration was determined using the Bradford method, and was expressed as mg/mL.

#### RNA preparation and quantitative real-time PCR of the placenta, fetal liver and fetal intestine

The placenta, fetal liver, and intestine were removed from the animals and were immediately snap-frozen in liquid nitrogen and ground to a fine powder in a mortar pre-cooled with liquid nitrogen. Total RNA was extracted by Trizol reagent (TRIzol Reagent, Takara). All the procedures were performed according to the manufacturer's protocols. RNA expression was determined using quantitative real-time PCR. The genes listed in Table 2 were amplified by cDNA specific primers (Invitrogen, USA) on a Thermal Cycler (CHRMO4-TM Thermal Cycler; Bio-Rad, Hercules, CA, USA), according to the manufacturer's instructions and the following protocol: 95°C for 10 s, 40 cycles at 95°C for 5 s; and 60°C for 25 s. Melt curve conditions ranged from 65 to 95°C, reading every 0.5°C and holding for 5 s every 0.5°C (temperature change velocity: 0.5°C). The relative expression levels of gene expression were normalized to those of the eukaryotic house-keeping gene, β-actin.

**Table 2:** The primer sequences of target genes and house-keeping gene.

Gene	Primers	Length	Gene Bank
Slc2a1	F:CCAGATACCTACCGGTTA	145	NM_138827.1
	R:TCCTTTAACTGCAGCTGA		
Slc2a3	F:CTGAAATGGAAAGGGTGT	112	NM_017102.2
	R:CCATGTCTTACACACAAGC		
Slc5a1	F:CATCCAGTCCATCACCAGTTACC	119	NM_013033.2
	R:ATCAGGAAGCCGAGAATCAGTC		
Slc7a1	F:TGTCCTGGTCTTGTGCTTC	133	NM_013111.2
	R:CCCTCCCTCACCGTATTTC		
Slc38a2	F:CCTTGGGCTTTCTTATGC	100	NM_181090.2
	R:GAGGAGGTGAACGGAGTA		
Slc38a4	F:GGTATCTTGGGCTTGTCC	125	NM_130748.1
	R:CCTTCCTTGGCTGTCTTC		
Pept1	F:GTGGGCTGAGTATGTTCTGTTCG	155	D50664.1
	R:TACGGGTTTTCCTTCCCTACG		
Apoa4	F:GCTCCATACCAAAGTCTCCACA	131	NM_012737.1
	R:TGCTTGTTCAGGTCTTCCAGAG		
Fabp2	F:GGAACAGAACTCACTGGGACCT	152	NM_013068.1
	R:CTTGGCCTCCACTCCTTCATA		
Fabp3	F: CAGGAAGGTCAAGTCGGTCGT	125	NM_024162.1
	R: TGGGTGAGAGTCAGGATGAGTT		
Lipase	F:GATGGACGGTGACAGGAATGTA	134	L03294.1
	R:CGGCAGACACTGGATAATGTTG		
MCT1	F:GCATTGGTGTCATTGGAGGTC	143	NM_012716.2
	R:GAGCCAGGGTAGAGAGGAACA		
MCT2	F:GAGAGAGAAAGCAAGGAGGAAG	123	NM_017302.2
	R:GTCTGAGGGAGGATTGTGTGTA		
MCT4	F:CATCATCACGGGCTTCTCCTAT	166	NM_030834.1
	R:AGCGGTTCACACACATACTGC		
GPx1	F:CTTCCCCAATCTGCCCTACTTA	112	NM_030826.3
	R:CTCCTCCTCTGTCTCTCCACAC		
Cu,Zn-SOD	F:GAACCAGTTGTGGTGTCAGGA	169	NM_017050
	R:CTCCAACATGCCTCTCTTCATC		
Mn-SOD	F:AGAGTTGCTGGAGGCTATCAAG	166	NM_017051
	R:CAGTGGGTCCTGATTAGAGCA		
Trx2	F:CGTACAATGCTGGTGGTCTAAC	110	NM_053331.2
	R:GTCTTGAAAGTCAGGTCCATCC		
CAT	F:CTCCCAACTACTACCCCAACAG	163	M11670.1
	R:CTCCTCCTCATTCAACACCTTC		
HIF-1a	F:CATCTCCACCTTCTACCCAAGT	110	AF057308
	R:GACTCTCTTTCCTGCTCTGTCTG		
JNK1	F:GCTACAAGGAGAACGTGGACTT	134	NM_016700.4
	R:GACAAGGTGTTCCGAGCTGTT		
P38	F:GCTGGTTTTGGACTCGGATAAG	168	U73142.1
	R:TTCATCGTAGGTCAGGCTCTTC		
Bax	F:GCGAATTGGAGATGAACTGGAC	161	NM_017059.1
	R:CTAGCAAAGTAGAAGAGGGCAACC		
Bcl-2	F:GGAGCGTCAACAGGGAGATGT	164	L14680.1
	R:GCCAGGAGAAATCAAACAGAGG		
β-actin	F:CACAGCTGAGAGGGAAAT	155	NM_045626.1
	R:TCAGCAATGCCTGGGTAC		

### Statistical Analyses

Data are presented as means and standard deviations. One-way ANOVA was used to analyze all data. A P-value of *<*0.05 was considered statistically significant.

## Results

### Reproduction Performance

Although the average daily feed intake and energy intake of SB group were significantly lower compared to all other groups (*P*<0.05, Table 3); however, the weight gain was not significant compared to the other groups (*P*>0.05). The fetal weight of the C group was significantly lower compared to the HF group (*P*<0.05), whereas there was no significant difference among the HF, SB, and CHC groups. Placental weight and placental efficiency were not affected by dietary energy types (*P*>0.05); however, the supplementation of SB increased the fetus number, which was a similar outcome to that observed in the HF group. Interestingly, abortion occurred more frequently in the CHC group; hence, it might contribute towards decreasing fetus number.

**Table 3:** Effects of diet on maternal performance*.

Parameters	C	HF	SB	CHC
NO. of Rats	16	19	19	15
Maternal body weight gain (g)	87.20±13.22	93.26±11.40	90.46±15.85	94.40±14.27
Average food intake (g/d)	20.06±3.02^c^	17.56±2.71^b^	14.80±2.24^a^	16.68±2.52^b^
Energy intake (kJ/d)	391±59^b^	389±60^b^	336±51^a^	369±56^ab^
Fetal weight (g)**	2.13±0.17^a^	2.29±0.11^b^	2.16±0.22^ab^	2.19±0.12^ab^
Placental weight (g)**	0.56±0.06	0.59±0.11	0.58±0.13	0.55±0.08
Fetus No**	10.67±1.23^a^	11.25±1.06^ab^	11.85±1.41^b^	10.71±1.44^a^
Abortion rate(%)	10	5	5	25

Note, C, control group; HF, high-fat and high-fiber group; SB, high-fat and sodium butyrate group; CHC, HF diet with CHC injection group.

*All data are means ± standard deviations; values in the same row with different letters are significantly different (P<0·05).

**Statistical analyses of fetal weight, placental weights and fetal numbers were based on the average value in each litter.

### SCFA Concentration in Cecal Content

Dietary fiber intake significantly increased SCFA concentrations in the cecal contents compared to the C and SB group (*P*<0.05, Table 4). The concentrations of acetate, propionate, and butyrate in the cecal content were lower in the SB group than in the HF and CHC groups (*P*<0.05). Yet, the ratio of butyrate was higher in the SB group than in the HF and CHC groups (*P*<0.05), whereas the ratios of acetate and propionate were similar (*P>*0.05). Interestingly, CHC injection significantly decreased the butyrate ratio compared to the HF group (*P*<0.05).

**Table 4:** Effects of diet on the content of SCFA in maternal cecum *.

Item	C	HF	SB	CHC
Acetate (µmol/g wet cecal contents)	10.04±3.60^a^	29.22±6.61^c^	10.01±2.66^a^	19.94±6.25^b^
Propionate(µmol/g wet cecal contents)	3.07±1.43^a^	14.50±5.13^c^	4.14±1.82^a^	8.72±3.74^b^
Butyrate (µmol/g wet cecal contents)	0.97±0.38^a^	1.72±0.55^b^	1.27±0.52^a^	1.93±0.92^b^
Tota SCFA (µmol/g wet cecal contents)	14.08±5.45^a^	45.43±10.47^b^	15.42±4.68^a^	30.59±10.46^b^
Acetate ratio (%)	72.29±5.19^b^	65.41±6.73^a^	65.83±5.52^a^	65.52±5.68^a^
Propionate ratio (%)	21.38±4.11^a^	28.20±5.60^b^	25.81±4.80^b^	30.87±5.56^b^
Butyrate rate (%)	6.33±2.90^b^	6.39±1.34^b^	8.36±2.20^c^	3.61±1.19^a^

Note, C, control group; HF, high-fat and high-fiber group; SB, high-fat and sodium butyrate group; CHC, HF diet with CHC injection group.

*Data are means ± standard deviations; values in the same row with different letters are significantly different (P<0·05).

### MDA and Protein Carbonyl Content

Although MDA content was significantly higher in the maternal serum of the SB group than in that of the other groups (*P*<0.05, Table 5), there were no differences between the SB and HF groups in the placenta (*P* = 0.092) or fetal liver. In addition, similar protein carbonyl levels were obtained in the HF, SB and CHC groups for the maternal serum, placenta, and fetus; however, these levels were higher compared to the C group (*P*<0.05).

**Table 5:** Effects of diet on the content of MDA and protein carbonyl*.

Items	C	HF	SB	CHC
MDA				
Maternal serum (nmol/ml)	1.50±0.16^a^	2.06±0.18^a^	3.79±0.95^c^	2.33±0.45^b^
Placenta(nmol/mg prot)	1.19±0.24^a^	1.03±0.17^a^	1.34±0.25^ab^	1.44±0.23^b^
Fetus liver (nmol/mg prot)	1.44±0.45^a^	1.87±0.34^b^	1.71±0.57^ab^	1.95±0.41^b^
Protein carbonyl				
Maternal serum (nmol/ml)	15.85±2.04^a^	16.61±1.40^ab^	17.69±1.65^b^	17.73±1.66^b^
Placenta (nmol/mg prot)	1.98±0.32^a^	2.68±0.55^b^	2.85±0.10^b^	2.48±0.48^b^
Fetus liver (nmol/mg prot)	5.94±0.61^a^	7.13±0.57^b^	6.75±1.16^b^	7.52±0.84^b^

Note, C, control group; HF, high-fat and high-fiber group; SB, high-fat and sodium butyrate group; CHC, HF diet with CHC injection group.

*Data are means ± standard deviations; values in the same row with different letters are significantly different (P<0·05).

### Antioxidant Enzymatic Activities

The GPx activity of the maternal serum, placenta, and fetal liver was lower in the SB group than in the HF group (*P*<0.05, Table 6). The maternal SB diet significantly decreased CAT activity in the maternal serum compared to the HF group (*P*<0.05). However, CAT activity in the placenta and fetal liver was similar in the SB, HF, and CHC groups (*P*<0.05), with no significant differences in the fetus liver among all 4 groups (*P*>0·05).

**Table 6:** Effects of diet on the activities of antioxidant related enzymes*.

Parameters	C	HF	SB	CHC
GPx				
Maternal serum (U/ml)	576.48±45.88^c^	594.16±51.49^c^	391.48±16.40^a^	444.94±23.85^b^
Placenta (U/mg prot)	67.03±8.73^ab^	78.21±10.50^b^	57.45±5.00^a^	55.74±11.35^a^
Fetus liver (U/mg prot)	74.97±8.43^a^	101.81±12.99^b^	82.93±9.98^a^	89.97±9.40^ab^
CAT				
Maternal serum (U/ml)	1.64±0.23^a^	3.51±0.30^b^	2.20±0.43^a^	2.30±0.85^a^
Placenta(U/mg prot)	9.23±1.28^b^	7.24±1.23^a^	6.35±1.34^a^	5.81±1.40^a^
Fetus liver (U/mg prot)	17.20±2.27	16.38±1.43	17.13±2.49	16.13±1.84
Maternal serum(U/ml)				
T-SOD	248.90±6.69	248.77±9.88	237.09±12.43	237.35±22.61
Cu,Zn-SOD	183.43±4.97^b^	181.06±5.89^b^	162.43±8.43^a^	184.74±9.96^b^
Mn-SOD	65.47±5.96^a^	66.39±5.50^a^	74.92±3.65^b^	62.59±4.16^a^
Placenta (U/mg prot)				
T-SOD	48.98±5.30^ab^	55.21±3.50^b^	44.25±4.37^a^	39.54±1.89^a^
Cu,Zn-SOD	32.83±4.43^b^	33.90±3.20^b^	20.48±2.47^a^	24.64±1.70^a^
Mn-SOD	16.15±3.73^a^	21.31±0.93^b^	23.76±2.48^b^	14.91±3.13^a^
Fetus liver (U/mg prot)				
T-SOD	44.89±2.44^a^	55.74±2.29^b^	44.25±1.10^a^	37.88±4.63^a^
Cu,Zn-SOD	20.08±2.16^b^	26.72±2.07^c^	13.57±3.06^a^	21.55±2.25^a^
Mn-SOD	24.81±1.48^a^	29.02±3.12^b^	30.68±2.19^b^	16.34±4.40^a^

Note, C, control group; HF, high-fat and high-fiber group; SB, high-fat and sodium butyrate group; CHC, HF diet with CHC injection group.

*Data are means ± standard deviations; values in the same row with different letters are significantly different (P<0·05).

A significant decrease in Cu-Zn SOD activity, in parallel to an increase in Mn-SOD, of the maternal serum in the SB group was observed (*P*<0.05, Table 6). However, significantly lower T-SOD and Cu-Zn SOD activity was recorded in the placenta of the SB and CHC groups compared to the HF group (*P*<0.05), while similar T-SOD and Cu-Zn SOD activity was recorded in the fetus liver and the placenta of the SB group.

### Placental and Fetus Liver Histology

**Figure 1:**
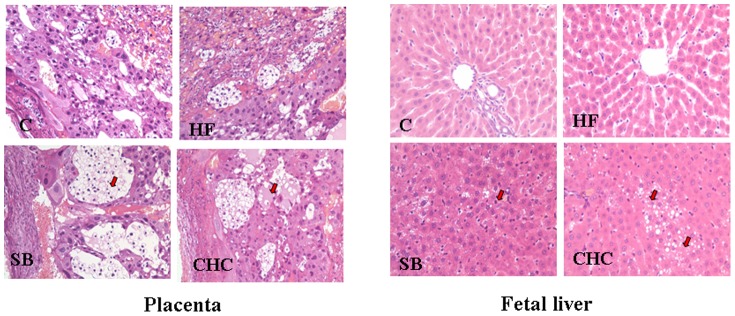
Effects of fiber on placenta (A) and fetal liver (B) structure (Magnification ×200). C, control group; HF, high-fat and high-fiber group; SB, high-fat and sodium butyrate group; CHC, HF diet with CHC injection group.

The placental strucutre and integrity in the HF group was similar to the control group (Figure 1). In the SB and CHC groups, the placental trophoblasts were clearly malformed, and were partially necrotic. In addition, CHC injection had an adverse impact on placental integrity. Furthermore, more fat deposits were observed in the fetal liver of the SB and CHC groups than in that of the HF group. In addition, the HF group exhibited better cellular structure and morphology compared to the SB and CHC groups.

### mRNA Expression Levels of MCT Genes in Maternal Cecum and Placenta

**Figure 2:**
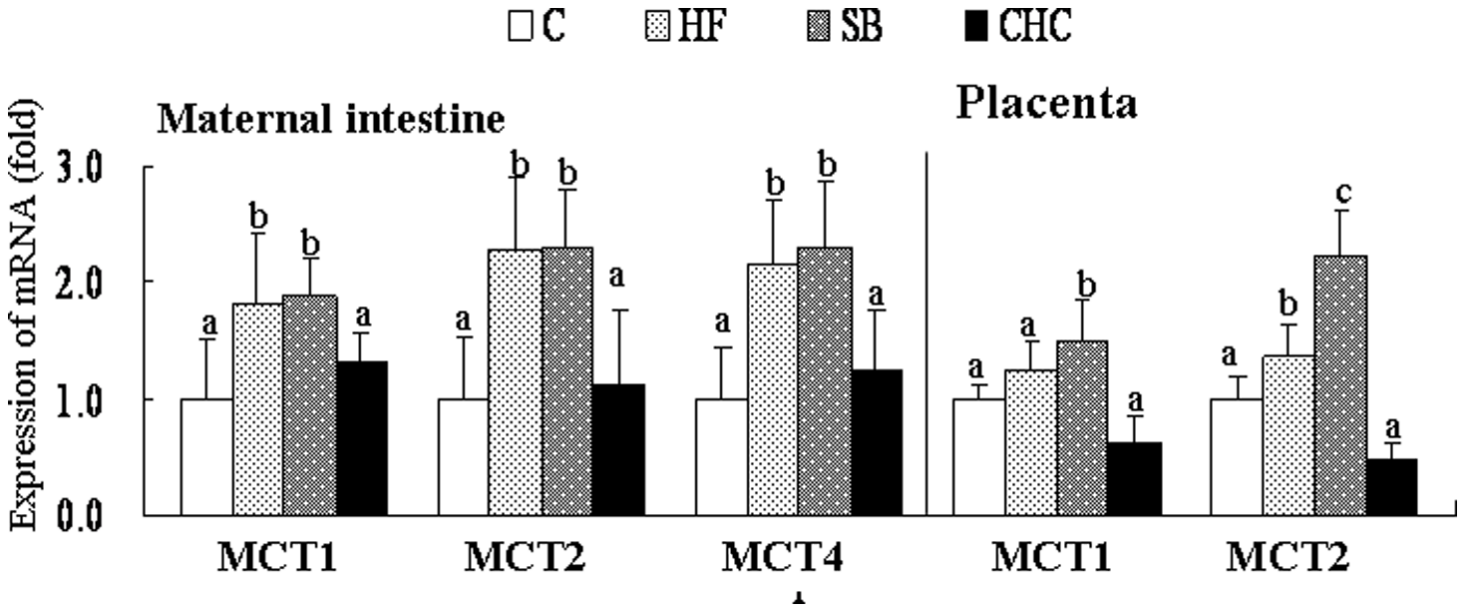
Effects of diet on MCTs mRNA expressions in maternal intestine and placenta. Values are means, with standard deviations represented by vertical bars (n = 6). a, b, c Mean values with unlike letters were significantly different (P<0·05). MCT1, Monocarboxylic acid transporters 1; MCT2, Monocarboxylic acid transporters 2; MCT4, Monocarboxylic acid transporters 4. C, control group; HF, high-fat and high-fiber group; SB, high-fat and sodium butyrate group; CHC, HF diet with CHC injection group.

The mRNA expression levels of MCTs in the maternal intestine are presented in Figure 2A. Compared to the C group, DF or SB supplement significantly increased the mRNA levels of MCT1, MCT2 and MCT4 expression in the maternal intestine (*P*<0.05), but the mRNA levels of MCT1, MCT2 and MCT4 of maternal intestine in the SB group were not different from the DF group. CHC injection significantly inhibited the expression of MCT2 and MCT4 of maternal intestine compared to the HF group (*P*<0.05). The mRNA levels of MCT1 and MCT2 of placenta in the SB group were higher than those in the DF group (*P*<0.05), although the MCT4 expression in placenta was not detected at this stage.

### mRNA Expression of Placental Nutrient Transporter Genes

**Figure 3:**
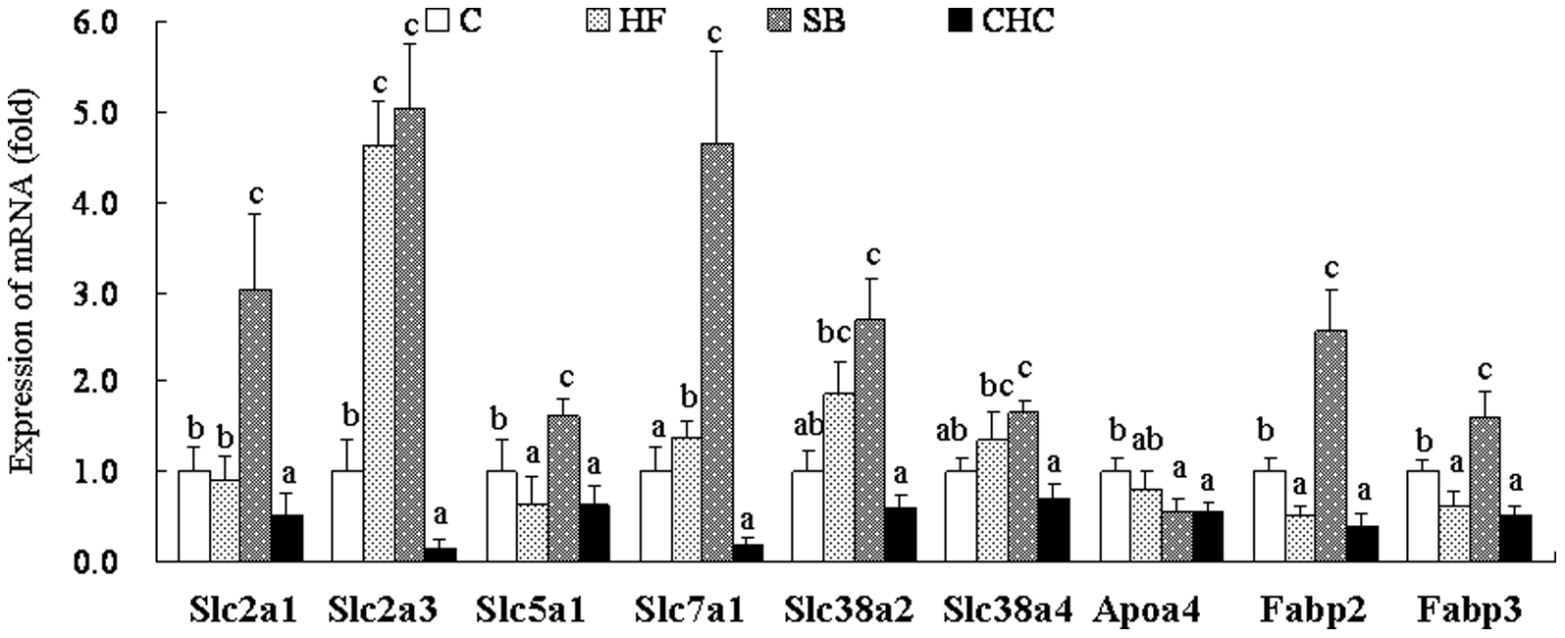
Effects of diet on mRNA expression of nutrients transporters in placenta. Values are means, with standard deviations represented by vertical bars (n = 6). a, b, c Mean values with unlike letters were significantly different (P<0·05). Slc2a1, glucose transporter 1; Slc2a3, glucose transporter 3; Slc5a1, sodium/glucose cotransporter 1; Slc7a1, cationic amino acid transporter 1; Slc38a2, system A amino acid transporter gene Slc38a2; Slc38a4, system A amino acid transporter gene Slc38a4; PepT1, peptide transporter 1; Apoa4, Apolipoprotein A4; Fabp2, fatty acid binding protein 2; Fabp3, fatty acid binding protein 3. C, control group; HF, high-fat and high-fiber group; SB, high-fat and sodium butyrate group; CHC, HF diet with CHC injection group.

The mRNA expression levels of placental nutrient transporter related genes are shown in Figure 3. We found that, compared to the C group, the HF diet significantly up-regulated the mRNA levels of the glucose transporter genes 3 (Slc2a3) and sodium/glucose cotransporter 1 (Slc5a1), and the system A amino acid transporter gene, Slc38a2; however, this diet also down-regulated the mRNA levels of the cationic amino acid, Slc7a1 (*P*<0.05). In comparison, no significant difference was obtained between the HF and SB groups regarding mRNA expression levels. Compared to the HF group, Slc38a4 and peptide transporter 1 (PepT1) were significantly downregulated after CHC injection. Interestingly, the mRNA expression of Apolipoprotein A4 (Apoa4) increased when DF was replaced with SB, whereas the mRNA expression of fatty acid binding protein 2 (Fabp2) and Fabp3 was significantly downregulated after CHC injection (*P*<0.05; Figure 3).

### mRNA Expression of Fetus Liver and Intestine

**Figure 4:**
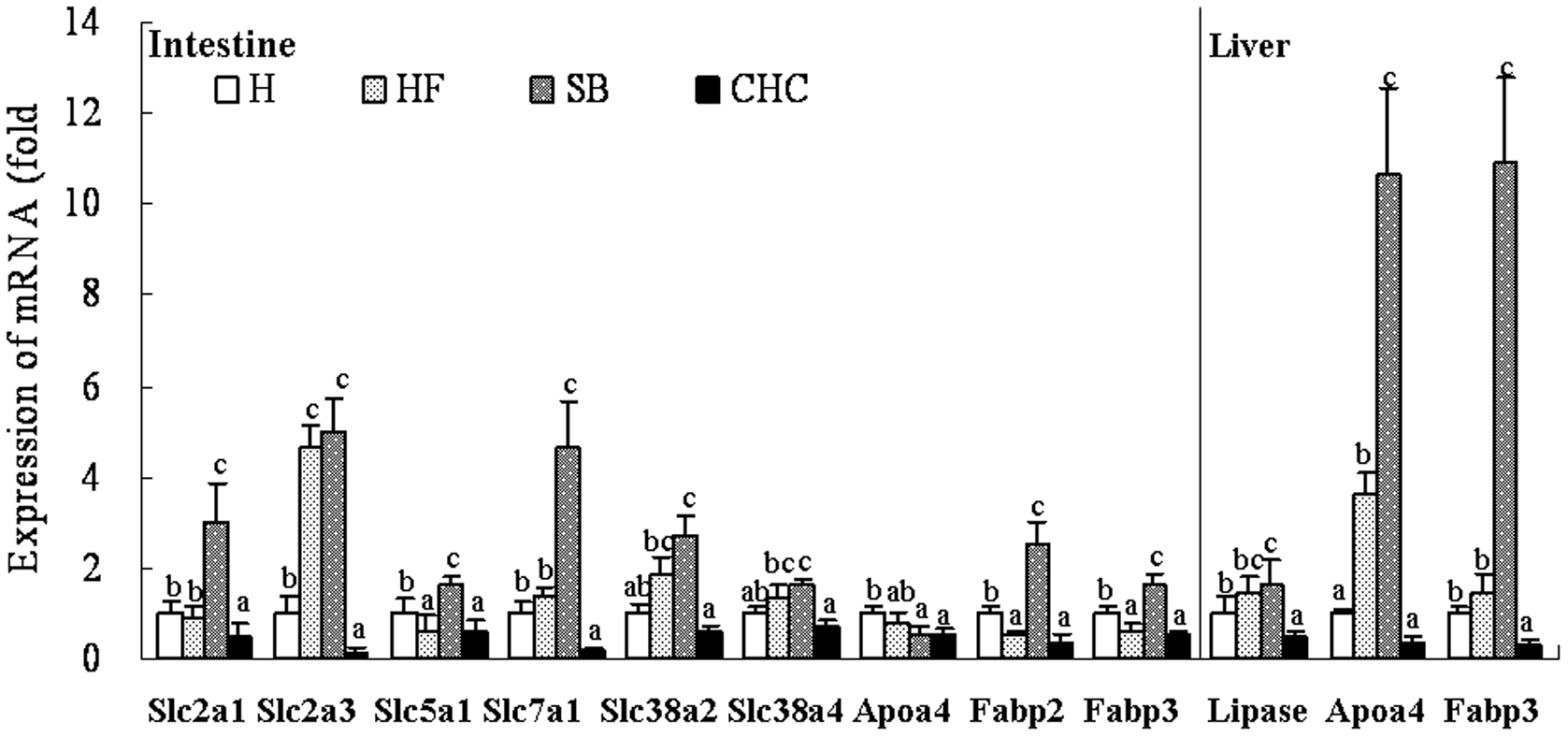
Effects of diet on mRNA expression of nutrients transporters genes in fetal liver (A) and fetal intestine (B). Values are means, with standard deviations represented by vertical bars (n = 6). a, b, c Mean values with unlike letters were significantly different (P<0·05). Lipase, lipase; Apoa4, Apolipoprotein A4; Fabp2, fatty acid binding protein 2; Fabp3, fatty acid binding protein 3; Slc2a1, glucose transporter 1; Slc2a3, glucose transporter 3; Slc5a1, sodium/glucose cotransporter 1; Slc7a1, cationic amino acid transporter 1; Slc38a2, system A amino acid transporter gene Slc38a2; Slc38a4, system A amino acid transporter gene Slc38a4. C, control group; HF, high-fat and high-fiber group; SB, high-fat and sodium butyrate group; CHC, HF diet with CHC injection group.

The fatty acid metabolism-related genes mRNA expression levels of the fetal liver are presented in Figure 4. On day 19 of pregnancy, Apoa4 and Fabp3 in the fetus liver were upregulated after DF was replaced with SB (*P*<0.05). In addition, Apoa4 mRNA expression in the fetus liver of the HF group was noticeably higher compared to the C group (*P*<0.05). CHC injection downregulated the expression of all detected genes compared to the HF group (*P*<0.05).

The mRNA expression of the fetal intestine was affected by maternal dietary intake (Figure 4). Compared to the HF group, the SB group showed a significant up-regulation of Slc2a1 and Slc5a1 mRNA levels (*P*<0.05). The expression of Slc7a1, Slc38a2 and Slc38a4 in the fetal intestine of the HF group was similar to the C group (*P*>0.05); however, the expression of these genes was, to some extent, lower compared to the SB group. Fabp2 and Fabp3 mRNA expression was more upregulated by maternal SB intake compared to the HF group (*P*<0.05), with no significant difference between the HF and CHC groups (*P*>0.05).

### mRNA Expression of Antioxidant-related Genes in Placenta and Fetus Liver

**Figure 5:**
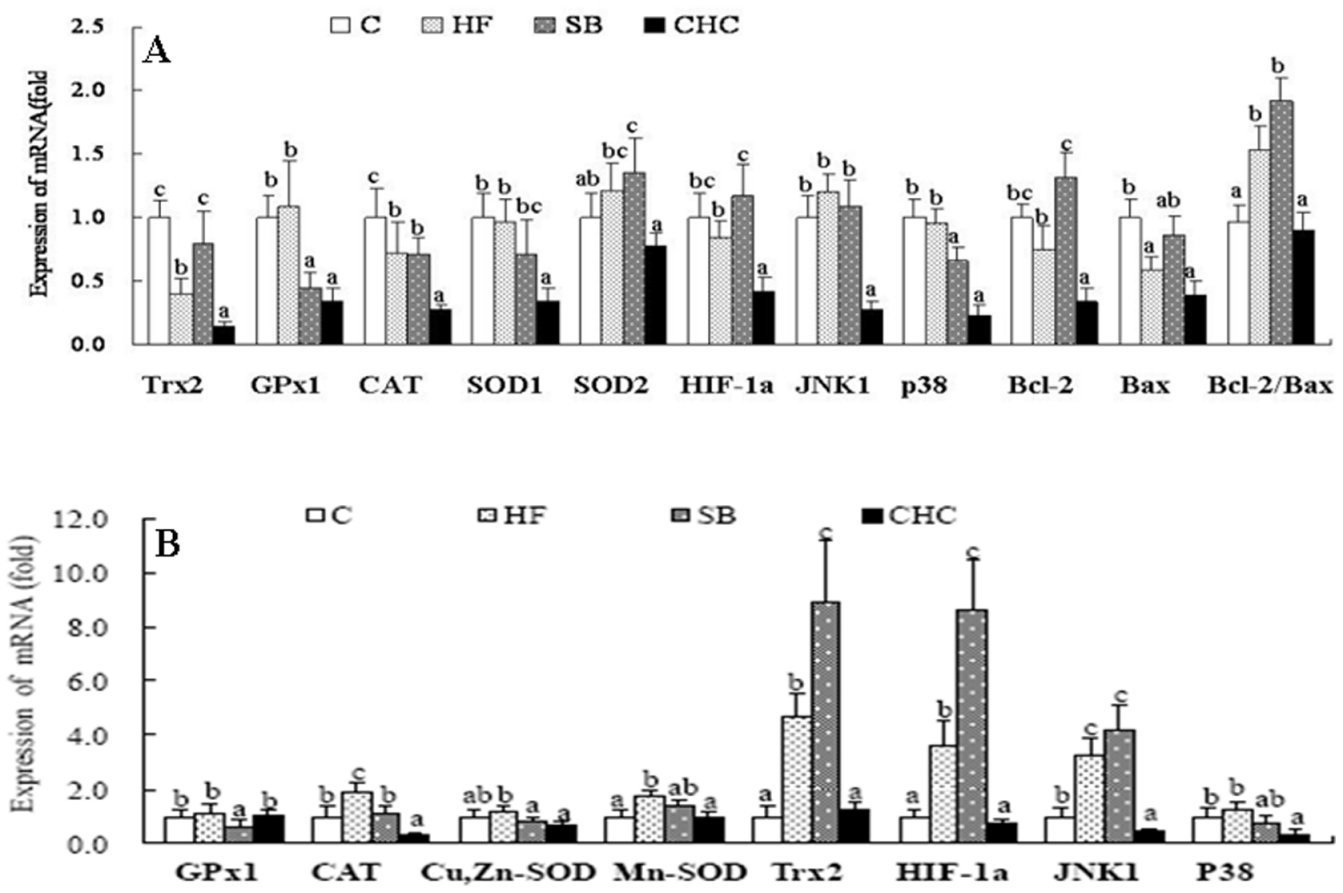
Effects of diet on mRNA expression of antioxidant-related genes in placenta (A) and fetus liver (B). Values are means, with standard deviations represented by vertical bars (n = 6). a,b,c Mean values with unlike letters were significantly different (P<0·05). Trx2, thioredoxin-2; GPx1, glutathione peroxidase 1; CAT, catalase; Cu,Zn-SOD, copper- and zinc-containing superoxide dismutase; Mn-SOD, manganese-containing superoxide dismutase; HIF-1a, hypoxia-inducible factor 1-a; JNK1, c-Jun N-terminal kinases; p38, p38; Bcl-2; Bax. C, control group; HF, high-fat and high-fiber group; SB, high-fat and sodium butyrate group; CHC, HF diet with CHC injection group.

The mRNA expression levels of antioxidant genes in the placenta and fetus liver are presented in Figure 5. There was no significant difference among genes between the C and HF groups, with the exception of the CAT gene (Figure 5A). In comparison, the mRNA levels of placental thioredoxin-2 (Trx2), GPx1, and Cu-Zn SOD were higher in the HF group than in the SB group (*P*<0.05). There was no difference in the mRNA expression of CAT and Mn-SOD between the HF and SB groups (*P*>0.05). CHC injection caused all genes detected in the placenta to be repressed compared to the HF group. Meanwhile, the mRNA expressions of hypoxia-inducible factor 1-a (HIF-1α) and Bcl-2 in the SB group were significantly higher compared to the HF group ((Figure 5B, *P*<0.05), and was similar to the C group (*P*>0.05). However, the mRNA expression of HIF-1a, c-Jun N-terminal kinases (JNK1), P38, and Bcl-2 were observed to be downregulated in the CHC group (*P*<0.05).

As shown in Figure 5B, DF supplementation increased the mRNA expression of CAT, Mn-SOD, Trx2, HIF-1a, and JNK1 (*P*<0.05). Significantly higher mRNA expression of GPx1, CAT, and Cu-Zn SOD was observed in the fetal liver of the HF group, while the mRNA expression of Trx2, HIF-1a, and JNK1 was lower in the fetal liver of the HF group compared to the SB group (*P*<0.05). However, there was no significant difference in the mRNA levels of HIF-1α, Trx1, and Cu-Zn SOD between the C and HF groups. Interestingly, the mRNA expression of all genes in the fetal liver was observed to be downregulated, with the exception of GPx1, after CHC injection (*P*<0.05).

## Discussion

SB is a short chain fatty acid that was originally identified as a product of the anaerobic bacterial fermentation of dietary fiber. This study confirmed that SB supplementation at 5% wt/wt in a high-fat diet has a similar effect to dietary fiber on embryo survival and abortion rate. In comparison, embryo survival decreased and the abortion rate increased when MCTs expression were inhibited by CHC injection, further validating the positive effects of fiber. However, research remains limited about the effects of SB and CHC injection on embryonic development. Previous studies have examined the effect of DF intake on reproductive performance and embryo development [4], [31]. Research has shown that the oral administration of SB increases the survival rate of diabetic rat pups by 8% [17]. SB treatment also increased the blastocyst formation rate of cloned pig embryos from 11.2% to 18.3% [12], while the survival of cultured embryonic stem cells also increased [14]. These results seem to suggest that SB might be e a substitute for DF towards improving reproductive performance, and appropriate SB intake in maternal model is needed to be explored in future.

The placenta is a very important organ for fetus survival and development, because it regulates the transport of nutrients and wastes. Our previous research demonstrated that a high-fat diet has an adverse effect on placental tissue integrity, with dietary fiber supplementation noticeably alleviating this phenomenon [3], [4]. In the present study, we confirmed that the supplementation of SB had a similar effect to HF at reducing the negative effect induced by a high-fat diet; however, this effect was inferior compared to DF. In particular, the placenta structure exhibited evident malformation and was partially necrotic after CHC injection, because MCTs were partially inhibited. This observation indicates that SCFA production may be involved in maintaining the integrity of placental tissue. In fact, the balance and imbalance of apoptosis and bax/bcl-2 affect the structure and function of the placenta [32]. The current study demonstrated that fiber supplementation of a high-fat diet decreased the expression of Bcl-2, whereas butyrate supplementation increased its expression, resulting in a higher Bcl-2/bax ratio. Recent studies have suggested that the up-regulation of Bcl-2 expression is responsible for an increase in the number of apoptotic renal cells [33]. In comparison, DF up-regulates the anti-apoptosis protein Bcl-2, and down-regulates the pro-apoptosis protein Bax [34], [35], resulting in a lower ratio of apoptosis [17]. Although it remains unclear how SB influences apoptosis, we can see that SB is not an efficient alternative to dietary fiber in maintaining placental structure integrity at this kind of model.

Of note, Bcl-2 exhibits antioxidant function and protects cells against mitochondrial oxidative stress [36]. In the current study, the expression of GPx1, Trx2, and Cu-Zn SOD in the placenta and GPx1, CAT, and Cu-Zn SOD in the fetal liver were lower in the SB group compared to the HF group; in contrast, Bcl-2 expression was higher in the SB group. This observed change in gene expression was supported by GPx1 and Cu-Zn SOD activity in the tissues and placenta structure after SB treatment. These results show that the anti-oxidant effect of SB is not as good as DF. It was previously reported that rats fed a high-fat diet suppressed Mn-SOD and Bcl-2 expression, leading to an increase in oxidative stress and the number of apoptotic renal cells [33]. DF supplementation of hamsters [35] or pregnant rats [4] ameliorated the oxidative stress induced by a high-fat diet. It has been previously demonstrated that butyrate and acetate reduce the induction of DNA damage by H_2_O_2_ in rat distal colon cells, whereas propionate was ineffective [37]. These findings imply the unique anti-oxidative effect of butyrate. In fact, butyrate beneficially influences oxidative stress in the colon of healthy humans [38], [39], in addition to alleviating oxidative stress induced by lipopolysaccharide in the intestinal cells [40] or streptozotocin diabetic rats [41], by changing the number of genes that is involved in radical scavenging [33], [39].

Reactive oxygen species (ROS) are potent inducers of various signaling pathways, encompassing the mitogen-activated protein kinases (MAPK) pathway and the Janus kinase/signal transducers and activators of transcription (JAK-STAT) pathway [42]. In the current study, HIF-1a mRNA was slightly upregulated in the placenta, while JNK1 and HIF-1a mRNA were slightly upregulated in the fetus liver. The supplementation of butyrate in a high-fat diet increases AMP kinase and p38 activity [43]. However, the effect of SB is eliminated after the knockout of JNK1, 2 stress-kinase genes [44]. Recent studies have found that β-hydroxybutyrate, which has a similar molecular structure to SB, suppresses oxidative stress in mice by increasing the transcription of encoding oxidative stress resistance factors, FOXO3A and MT2, and by increasing histone acetylation at the Foxo3a and Mt2 promoters [45]. Whether maternal SB intake has this effect on offspring requires future study.

Interestingly, although the SB supplementation of high-fat diets improves maternal, placental, and fetal antioxidant capacity, its effect is lower compared to various DFs, such as wheat bran and oat, which is consistent with the results of Kumar et al. [18]. This phenomenon is probably because wheat bran and oat are fermentable, producing butyrate, in addition to relatively high amounts of propionate and acetate. In fact, different DFs have different effects on metabolism because of different fermentable capacities and fermentation products [46]. In addition, fiber facilitates the excretion of dietary fat into the feces, which greatly reducing the oxidative stress induced by high-fat diets [47], whereas this effect is very limited for SB. Of note, it is possible that butyrate plays a major role in SCFA mechanisms, and it cannot completely substitute DF; however, information remains limited on this subject.

Compared to DF intake, SB has similar effects at up-regulating glucose and amino acid transporters; therefore, SB affects embryo growth and development. Research about DF and its metabolism, such as SB, on fetus development and transporters remains limited. In vitro cell culture, SB treatment had no effect on GLUT-1 mRNA expression, but upregulated GLUT2 mRNA expression by activating specific regions within the human GLUT2 promoter [48], [49]. The system A amino acid transporters, Slc38a2 and Slc384, mediate the transport of neutral amino acids, and are modulated by a butyrate-sensitive mechanism [50]. Meanwhile, butyrate increases PepT1 mRNA and protein expression and activity at the transcriptional level in colonic epithelial cells [51]. Of note, in the current study, the fatty acid transporters in the SB group were quite different to those of the HF and CHC groups in the placenta and fetus liver. This observation indicates that offspring might have a different fat metabolism. Apoa4 is an anorectic gene and involved in various activities, such as antioxidant activity and lipoproteins metabolism. Interesting, the expression of Apoa4 was up-regulated after SB addition which may alleviate the negative effect of high-fat diet induced obesity. In fact, butyrate alleviates metabolic stress in pregnant obese mice without any obvious toxicity to the fetus [17]. In butyrate-treated mice, the phosphorylation of AMP-activated protein kinase (AMPK) and p38, and the peroxisome proliferator-activated receptor (PPAR)-δ increased in the skeletal muscle, which alters the energy metabolism and, hence, the fat content (body weight) of obese mice was reduced [43]. Piglets born to sows that were supplemented with butyrate had 15% higher body weight, which was related to fatty acid metabolism [52]. In fact, the expression of lipoprotein lipase, fatty acid synthase, and PPAR-γ mRNA [53] was upregulated in propionate-infused sheep. SCFA-supplemented total parenteral nutrition up-regulates jejunal GLUT2 mRNA and ileal GLUT2 abundance, as well as increasing the jejunal uptake of L-glucose and lauric acid [54].

The current study indicates that the maternal intake of SB not only affects the expression of placental nutrient transporter gene, but also has a major effect on the gene expression of the fetal liver and intestine. Recent studies have shown that a total of 19400 genes respond to butyrate in a human colonic epithelial cell line [55], indicating that SB plays an important role in the regulation of gene expression and tissue homoeostasis. Although the effects of SB on gene expression have yet to be clarified, butyrate is an essential agent for determining the role of histone acetylation in chromatin structure and function; thus, SB might influence embryo development and gene expression [56]. Inhibition of histone deacetylases represents one of the mechanisms of GST activation by butyrate, which explains the possible role of butyrate on antioxidant related gene expression [57]. To the best of our knowledge, the imprint gene IGF2 plays an important role on placenta transporter expression and fetus growth, with butyrate being involved in the induction of Igf-2 activation and regulation of gene expression by histone modification [58].

In summary, the use of sodium butyrate as an alternative to dietary fiber in high-fat diets produces similar effects on reproduction performance. Maternal fiber intake was clearly superior to SB with respect to structure integrity of placenta and fetus liver, and redox regulation. In addition, DF regulated gene expression in the fetus liver and intestine very differently to SB. These results indicate that butyrate alone cannot completely replace dietary fiber in high-fat diet with regard to improving embryo development and anti-oxidant capacity.
